# Developing tools for evaluating inoculation methods of biocontrol *Streptomyces* sp. strains into grapevine plants

**DOI:** 10.1371/journal.pone.0211225

**Published:** 2019-01-24

**Authors:** Sandra González-García, Jose Manuel Álvarez-Pérez, Luis E. Sáenz de Miera, Rebeca Cobos, Ana Ibañez, Alba Díez-Galán, Enrique Garzón-Jimeno, Juan José R. Coque

**Affiliations:** 1 Instituto de Investigación de la Viña y el Vino, Universidad de León, León, Spain; 2 Área de Genética, Departamento de Biología Molecular, Universidad de León, León, Spain; 3 RGA Bio-Investigación S.L., León, Spain; Colorado State University, UNITED STATES

## Abstract

The endophytic *Streptomyces* sp. VV/E1, and rhizosphere *Streptomyces* sp. VV/R4 strains, isolated from grapevine plants were shown in a previous work to reduce the infection rate of fungal pathogens involved in young grapevine decline. In this study we cloned fragments from randomly amplified polymorphic DNA (RAPD), and developed two stably diagnostic sequence-characterized amplified region (SCAR) markers of 182 and 160 bp for the VV/E1 and VV/R4 strains, respectively. The SCAR markers were not found in another 50 actinobacterial strains isolated from grapevine plants. Quantitative real-time PCR protocols based on the amplification of these SCAR markers were used for the detection and quantification of both strains in plant material. These strains were applied on young potted plants using two methods: perforation of the rootstock followed by injection of the microorganisms or soaking the root system in a bacterial suspension. Both methods were combined with a booster treatment by direct addition of a bacterial suspension to the soil near the root system. Analysis of uprooted plants showed that those inoculated by injection exhibited the highest rate of colonization. In contrast, direct addition of either strain to the soil did not lead to reliable colonization. This study has developed molecular tools for analyzing different methods for inoculating grapevine plants with selected *Streptomyces* sp. strains which protect them from fungal infections that enter through their root system. These tools are of great applied interest since they could easily be established in nurseries to produce grafted grapevine plants that are protected against fungal pathogens. Finally, this methodology might also be applied to other vascular plants for their colonization with beneficial biological control agents.

## Introduction

Young grapevine decline (YGD) is a major threat to the wine and grape industry, especially since the 1990s when it entered a period of rapid expansion. Many factors are involved in YGD, although it is accepted that the fungal pathogens responsible for grapevine trunk diseases (GTDs) are one of the major culprits [1]. The main GTDs associated with YGD are Petri disease, primarily caused by *Phaeomoniella chlamydospora* and several species of the genus *Phaeoacremonium* [1], and black-foot disease, caused by different species belonging to the genera *Campylocarpon*, *Cylindrocladiella*, *Dactylonectria* and *Ilyonectria* [2, 3]. Most of these pathogens can penetrate the plant through the root system [1, 4, 5], and therefore, the presence of endogenous pathogens in grafted plants (planting material) produced in nurseries is also highly frequent. This infection is due either to prior infection of the parent plants from which the rootstocks are obtained, or contamination occurring during the propagation process [1]. Accordingly, there has been a growing demand among winemakers for methods to control fungi causing GTDs that infect the plant through the root system, and for reducing the infection rate of planting material produced in nurseries. Unfortunately, there is not much information available about this subject. Some nursery trials have shown that application of different *Trichoderma* strains to soils or the grapevine root system had a growth-stimulating effect and reduced the infection rate of *Cylindrocarpon* sp., *Phaeoacremonium* sp., and *P*. *chlamydospora* [5, 6]. Another study involving the application of *Trichoderma harzianum* at different vine-growth stages in a nursery indicated that its application during the rooting stage showed some efficacy in protecting against *P*. *chlamydospora* infections. However, an increase in vine mortality was observed at the end of the growing season [7].

Recently, several actinobacterial strains isolated and characterized from the rhizosphere, or the inner tissues of the root system of young grapevine plants, exhibited antifungal activity against fungi that cause YGD [8]. Field trials carried out in a nursery led to the identification of one endophytic strain, *Streptomyces* sp. VV/E1, and two rhizosphere isolates, *Streptomyces* sp. VV/R1 and *Streptomyces* sp. VV/R4, which significantly reduced the infection rate produced by the fungal pathogens *Dactylonectria* sp., *Ilyonectria* sp., *P*. *chlamydospora* and *Phaeoacremonium minimum* involved in YGD [8]. In the current study, SCAR (sequence characterized amplified region) markers and qPCR protocols for the *Streptomyces* sp. VV/E1, and *Streptomyces* sp. VV/R4 strains were developed to specifically detect and quantify their presence in plant material. These molecular tools allowed us to analyse the effectiveness of different inoculation methods of young grapevine plants with these selected strains, as well as to determine the presence of these strains inside the root system of the inoculated plants.

## Materials and methods

### Microbial strains, culture media and growth conditions

*Streptomyces* strains VV/E1 and VV/R4 were isolated from the root system (endophytic) and the rhizosphere of young grapevines, respectively [8]. Both strains were grown in tryptic soy broth (TSB) medium (Sigma-Aldrich, St. Louis, Missouri, USA) at 220 rpm and 30°C for 3 days.

### Isolation of genomic DNA

Genomic DNA of both strains was isolated from liquid cultures grown in TSB medium (Sigma-Aldrich) as described by Hopwood *et al*. [9]. DNA was checked by electrophoresis in a 0.8% (w/v) agarose gel. DNA concentration was measured using a NanoDrop 2000 spectrophotometer (Thermo Fisher Scientific, Waltham, Massachusetts, USA) and stored at -20°C.

### Amplification of random polymorphic DNA sequences (RAPD)

Thirteen random primers, 10-mer each, were used for developing RAPD-PCR protocols in order to identify the differential polymorphism of 10 different actinobacteria (VV/E1-VV/E5 and VV/R1-VV/R5) [8] according their electrophoretic band patterns. Primers used were: OPA2 (5´-TGCCGAGCTG-3´), OPA9 (5´-GGGTAACGCC-3´), OPA10 (5´-GTGATCGCAG-3´) [10], P1 (5´-GGTGCGGGAA-3´), P2 (5´-GTTTCGCTCC), P3 (5-GTAGACCCGT-3´), P4 (5´-AAGAGCCCGT-3´), P5 (5´-AACGCGCAAC-3´) [11], R1 (5´-CACGCCCTTC-3´), R3 (5´-ATGCAGCCAC-3´) [12], SS1 (5´- GTCAACGCGG-3´), SS2 (5´- AACGGCTCGC-3´) and SS3 (5´- GAGATCGCGC-3´). Amplification reactions were developed in 1X paq5000 buffer (Agilent Technologies, Santa Clara, California, USA) with 625 nM primer (IDT, Leuven, Belgium), 0.125 nM dNTP mix (EURx, Gdansk, Poland), 1 U Paq5000 DNA polymerase (Agilent Technologies) and 30 ng template DNA. Nuclease-free water was used to bring the total reaction volume to 20 μL. PCR amplification was carried out in 0.2 mL sterile tubes using a Mastercycler Gradient thermocycler (Eppendorf, Hamburg, Germany). PCR conditions for primers R1, R3, OPA2, OPA9 and OPA10 were: 94°C for 3 min; 40 cycles of 94°C for 60 s, 40°C for 60 s, 72°C for 90 s; and a final extension of 72°C for 10 min. PCR conditions for the rest of the primers were: 94°C for 3 min; 45 cycles of 94°C for 30 s, 36°C for 30 s, 72°C for 60 s; and a final extension of 72°C for 10 min. Tubes were kept at 4°C until the reactions were analysed in 1.5% agarose gels in 1X TAE buffer containing 0.15 μg/mL ethidium bromide (Sigma-Aldrich) and photographed using VWR GenoSmart equipment (VWR, Barcelona, Spain).

### Cloning and sequencing of DNA fragments

Two distinctive bands of 916 (VV/E1 strain) and 1862 bp (VV/R4 strain) were excised from the gel and the DNA was recovered and purified using freeze-squeeze [13]. Purified DNA was ligated into pBluescript (II) KS (+) (Agilent Technologies) using T4 DNA ligase (Fermentas, Glen Burnie, Maryland, USA) and transformed into *E*. *coli* DH5α competent cells (Thermo Fisher Scientific). Recombinant clones were selected on LB agar plates (Sigma-Aldrich) containing 50 μg/mL ampicillin, 50 μg/mL X-Gal, and 50 μg/mL IPTG. Blue-white screening was used and white colonies were checked for the presence of the correct insert using a double digestion with *Eco*RI–*Sal*I restriction enzymes (EURx). The DNA was then sequenced using universal primers T7 (5´-TAATACGACTCACTATAGGG-3´) and M13 reverse (5´-CCTTTGTCGATACTGGTAC-3´) [14]. Sequence data were analysed using BLASTx (Basic Local Alignment Search Tool program) (https://blast.ncbi.nlm.nih.gov/).

### Amplification of SCAR markers

The nucleotide sequence of each of the cloned RAPD fragments was used to design pairs of SCAR primers targeting poorly conserved regions or intergenic regions. The paired primers, SCAR.E1_fw (5´-GGTTCACGGTATCTGTTTACTCACC-3´) and SCAR.E1_rv (5´-GTTGGTTGAACCCTTCTTCCG-3´) were used for amplification of a VV/E1 SCAR marker, while primers SCAR.R4_fw (5´-CCGAAGGGCTCTCTGAGTTGC-3´) and SCAR.R4_rv (5´-CTTCGCCGCTGAGGCACG-3´) were used for the VV/R4 strain. SCAR amplification from genomic DNA samples was performed in a reaction containing 1X paq5000 buffer (Agilent Technologies), 0.5 μM each of the primers (IDT), 0.1 mM dNTP mix (EURx), 1 U Paq5000 DNA polymerase (Agilent Technologies), and 30 ng genomic DNA. Nuclease-free water was used to bring the reaction volume to 25 μL. PCR conditions were as follows: 95°C for 2 min; 30 cycles of 95°C for 30 s, melting temperature (Tm) for 30 s, 72°C for 40 s; with a final extension of 72°C for 7 min. The Tm for SCAR.E1_fw/SCAR.E1_rv primers was 60°C, and 63°C for the SCAR.R4_fw/SCAR.R4_rv primers. Tubes were kept at 4°C until analysis on 1.5% (w/v) agarose gels (as described above). In order to check specificity of the 2 sets of primers and the SCAR marker developed, genomic DNA from 50 actinobacterial strains isolated from rhizospheres and the root systems of young grapevine plants [8] were used as templates in a conventional PCR under the same conditions.

### Quantitative SYBR Green real-time PCR (qPCR) assays

The qPCR technique [15] was developed based on the above mentioned SCAR primers. The reaction mixture contained 1X SYBR Green master mix (Takara, Shiga, Japan), 0.2 μM of each primer and 5 μL bacterial DNA isolated from axenic cultures of VV/E1 and VV/R4 strains as templates. Nuclease-free water was used to bring the reaction volume to 20 μL. VV/E1 bacterial DNA, ranging in amounts from 5.65 x 10^6^ fg to 5.65 x 10^2^ fg per reaction, and 5.2 x 10^6^ fg to 5.2 x 10^2^ fg per reaction for VV/R4 strain, were used. Amplification was performed in qPCR 96-well plates (Agilent Technologies), and run in the Mx3005P qPCR System (Agilent Technologies). Thermal conditions were: 95°C for 2 min, 40 cycles of 95°C for 20 s, elongation at Tm for 20 s, followed by 1 cycle of 95°C for 1 min, 55°C for 30 s and 95°C for 30 s. Tm was the same as for the conventional PCR. All analyses were performed as three independent experiments with three replicates for each dilution.

### Sensitivity of actinobacterial detection in grapevine wood

It was determined by calculating qPCR standard curves using 10-fold serial dilutions of genomic DNA (5.65 x 10^6^ fg to 5.65 x 10^2^ fg DNA were used for *Streptomyces* sp. VV/E1, whereas 5.2 x 10^6^ fg to 5.2 x 10^2^ fg were used for *Streptomyces* sp. VV/R4). Nuclease-free water was used as non-template control (NTC). The limit of quantification (LOQ) was estimated according to the coefficient of variation [*CV* = 100 x (*SD*/*mean value*)] of back-calculated concentrations (5 replicates) of genomic DNA from both *Streptomyces* strains: a *CV* below 35% was assumed to be the LOQ (S2 Fig) [16]. To ascertain whether the primer sets designed for the amplification of SCAR markers were reliable for the *Streptomyces* sp. PCR detection in grapevine wood, without the interference of any grapevine genomic DNA, quantitative real-time assays were carried out in the presence of grapevine genomic DNA. Each PCR mixture contained 5.2 ng of *Streptomyces* sp. VV/E1 genomic DNA, or 5.7 ng of *Streptomyces* sp. VV/R4 genomic DNA, serially diluted tenfold up to 10^−5^. The same reactions were supplemented with 5.2 and 5.7 ng grapevine genomic DNA. The final ratio of actinobacteria-to-grapevine genomic DNA ranged from 1:1 to 1:100,000. The experiments were conducted twice with three replicates each.

### Calculation of the correlation between quantity of genomic DNA from *Streptomyces* sp. VV/E1 and VV/R4 strains and number of cells

Both SCAR markers were amplified by conventional PCR, purified using the NucleoSpin Gel and PCR Clean-up kit (Macherey-Nagel) and quantified on NanoDrop. Since the size and nucleotide composition of each amplicon are known, the number of copies of each DNA template (SCAR copy numbers) were easily estimated using an online calculator (http://cels.uri.edu/gsc/cndna.html). Thus, PCR products were serially diluted 10-fold and used as templates to generate qPCR standard curves, one for each SCAR marker (S3A Fig). Genomic DNA quantified on NanoDrop were also serially diluted 10-fold and the copy number of each SCAR was estimated using the previous standard curves (S3B Fig). Thus, the number of cells (cells/mg of grapevine wood) could be directly estimated through the additional correlation between genomic DNA quantity and the corresponding number of copies of each amplicon (S3C Fig). qPCR conditions were the same as described above.

### Inoculation of grapevine plants with *Streptomyces* strains

Sixty 1-year-old *Vitis vinifera* (cv. Tempranillo) plants grafted on Richter 110 (110R) rootstock were supplied by Viveros Villanueva Vides S.L. (Larraga, Spain). Their root systems were uniformly trimmed to a length of 10 cm, and immediately inoculated with *Streptomyces* sp. VV/E1 or *Streptomyces* sp. VV/R4 strains [8], and planted in plastic pots containing 50 L of Compo Sana 20-20-30 commercial substrate mixture, which is suitable for grapevines (Compo Sana, Barcelona, Spain). Potted vines were grown outdoors under shade-cloth and regularly irrigated with tap water. Experimental potted vineyards were developed in the facilities of the Instituto de Investigación de la Viña y el Vino (Universidad de León, Spain; 42°35'01.0"N 5°35'20.2"W).

**Fig 1:**
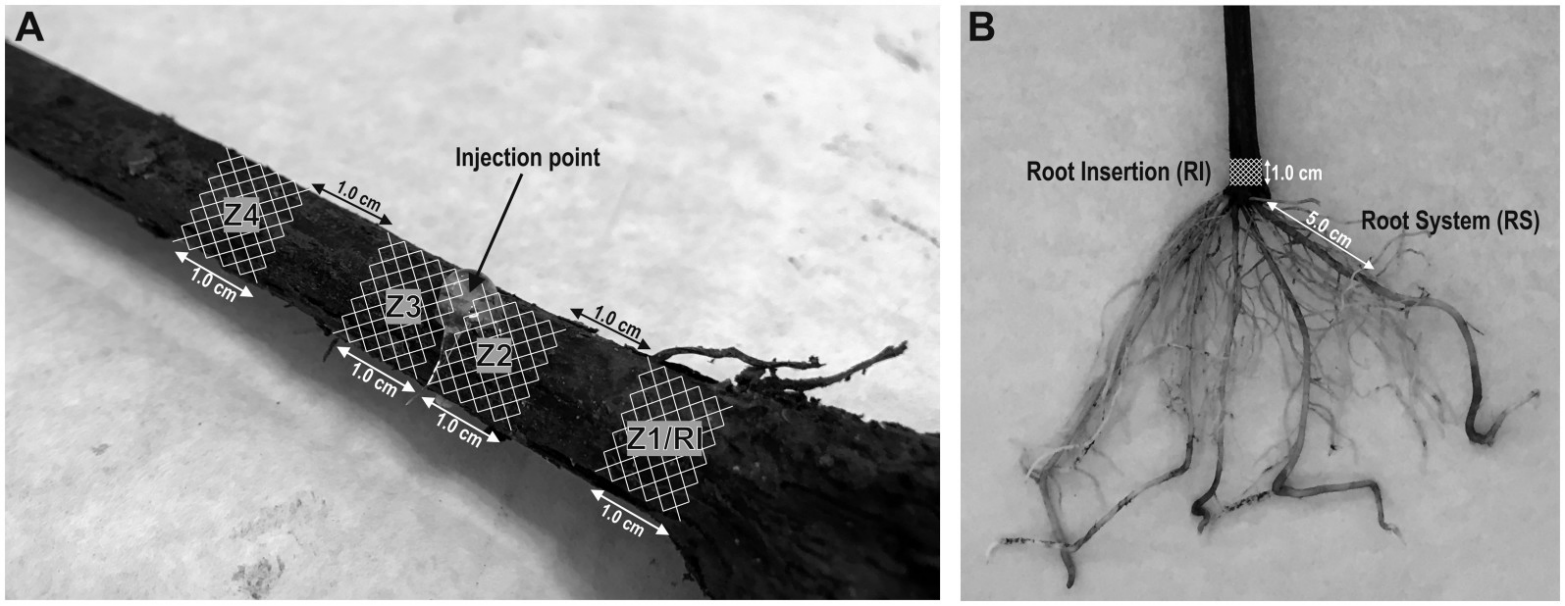
Scheme of inoculation of grapevine plants and areas analysed. Image of (A) the inoculation point in the rootstock of plants treated by injection, as well as the four 1.0 cm cylinders (Z1/RI, Z2, Z3 and Z4) analysed once the plants were uprooted, and (B) the zones defined as root insertion point (RI) and root system (RS) also analysed in plants inoculated by immersion in a cell suspension. Note that Z1/RI and RI zones in plants inoculated by injection or immersion are equivalent for analytical purposes.

For each *Streptomyces* sp. strain, 6 different batches were processed as follows: batch 1 consisted of non-inoculated plants (control); batch 2, plants inoculated by immersion of the root system for 24 hours in a bacterial suspension (5 x 10^5^ cells/mL); batch 3 was identical to batch 2, but an additional booster treatment was applied 30 days after planting. The booster treatment was added to the soil into a 15 cm hole made using a hand drill in parallel to the rootstock. In this way the bacteria (10^7^ cells; 20 mL of 5 x 10^5^ cells/mL) were applied directly to each pot in the proximity of the root system. Batch 4 plants were inoculated by direct injection of bacteria into the rootstock, (50 μL of a bacterial culture containing 5 x 10^5^ cells) 3 cm above the root insertion point (RI) via a pipette placed in a drilled hole (5 mm deep) (Fig 1A). Batch 5 was like batch 4, but with an additional booster treatment applied (similar to treatment 3). Batch 6 plants were an additional control. They were not inoculated by either method, although the same booster treatment was applied 30 days after planting in order to observe the effect of direct actinobacteria addition to the soil. Plants were uprooted and analysed 180 days after potting.

### Analysis of the experimental grapevines planted in pots: Detection and quantification of *Streptomyces* sp. VV/E1 and VV/R4 strains in wood samples

Once uprooted, samples from all the plants were taken from the root system (RS), and the root insertion point (RI) (Fig 1B). Plants from treatment groups 3 and 4 (inoculated by injection) were subjected to a more detailed analysis by zones (Fig 1A). Zone Z1 was located 2–3 cm below the inoculation point; zone Z2 was 0–1 cm below the inoculation point, while zones Z3 and Z4 were 0–1 cm and 2–3 cm above the inoculation site, respectively. 1 cm wood fragments were surface-sterilized [17], cut into small pieces using a sterile scalpel, frozen in liquid N_2_ and manually-ground up with a mortar into a very fine powder. DNA was extracted from 50 mg of dry weight material using a Nucleo Spin Plant II Extraction kit (Macherey-Nagel). DNA concentration was estimated using a NanoDrop and stored at -20°C. *Streptomyces* sp. VV/E1 and VV/R4 strains were detected and quantified by qPCR amplification of the developed SCAR markers, as indicated above.

#### Data analysis

Statistical analyses were performed with R software (ver. 3.3.1; https://www.r-project.org/). Analysis of variance was explored with linear models and level of significance between treatment groups was determined by an LSD-test (least significant difference) from the Agricolae R package (ver. 1.2–8, 2017) [18].

## Results

### Identification and characterization of differential RAPD amplification fragments of *Streptomyces* sp. VV/E1 and *Streptomyces* sp. VV/R4

**Fig 2:**
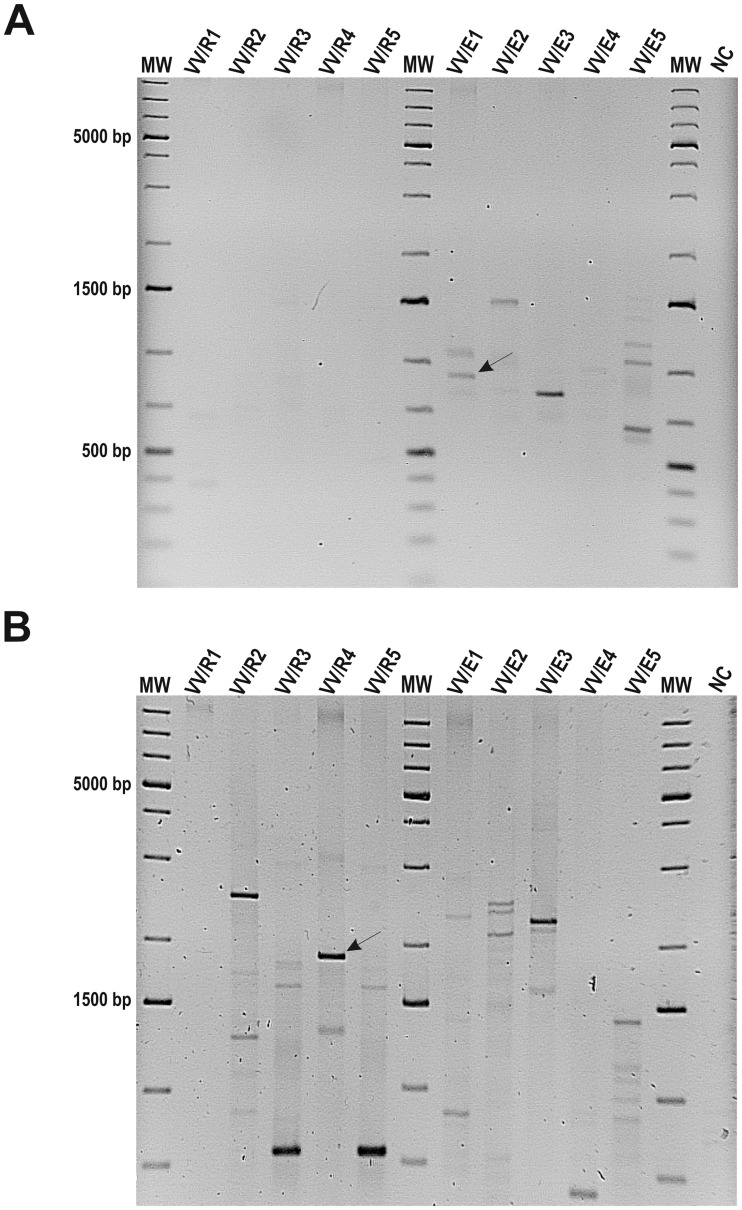
RAPD profiles. RAPD profiles generated by (A) P4 primer and (B) OPA2 primer for rhizosphere (VV/R1 to VV/R5) and endophytic (VV/E1 to VV/E5) *Streptomyces* sp. strains isolated from the root system of young grapevine plants. MW: GeneRuler 1kb DNA Ladder Plus (Thermo Fisher Scientific). Specific bands amplified from isolates VV/E1 and VV/R4 that were selected for SCAR markers design are indicated by arrows. NC (negative control).

Different electrophoretic band patterns of 5 *Streptomyces* sp. rhizosphere strains (VV/R1 to VV/R5), and 5 actinobacterial endophytic strains (VV/E1 to VV/E5) [8] were obtained by amplification of RAPD sequences using up to 13 different primers (S1 Fig). A 916 bp band was uniquely amplified from *Streptomyces* sp. VV/E1 strain using the P4 primer. Similarly, a 1862 bp band was amplified from only *Streptomyces* sp. VV/R4 using the OPA2 primer (Fig 2). Both DNA bands were purified from agarose gels, cloned, sequenced and compared with those already present in GenBank database.

**Fig 3:**
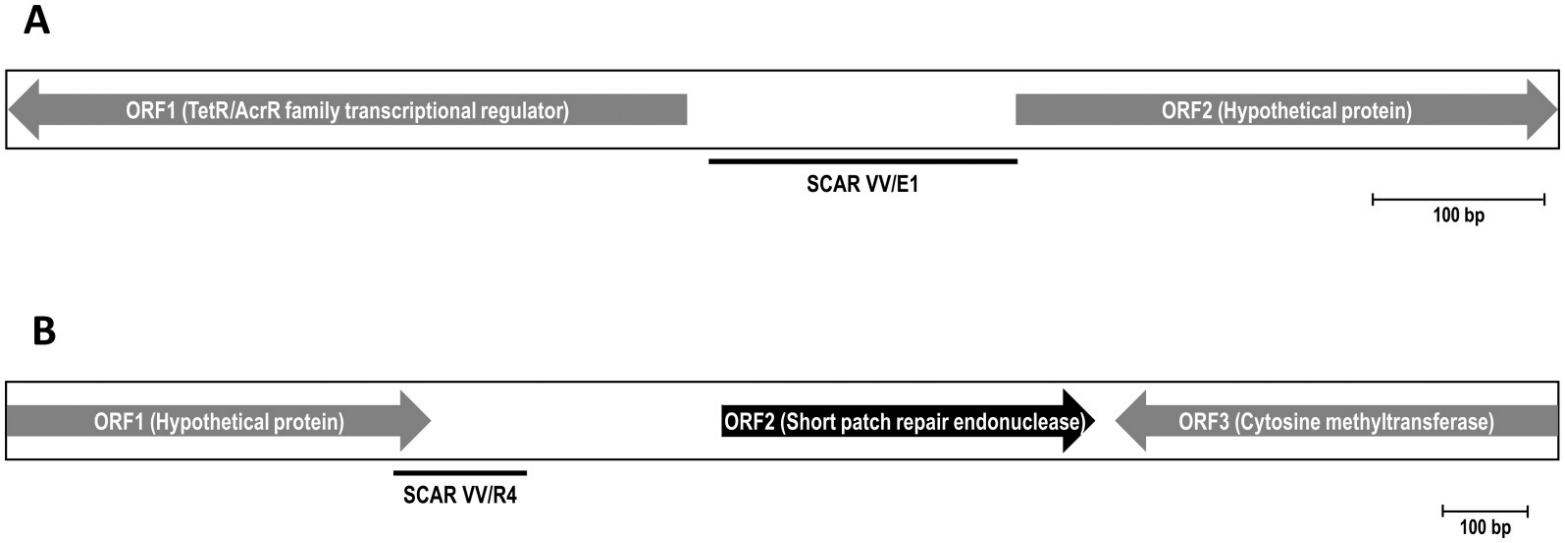
Representative maps of the characterized RAPD fragments. (A) *Streptomyces* sp. VV/E1 and (B) *Streptomyces* sp. VV/R4. Incomplete ORFs are indicated by grey arrows, whereas black arrows correspond to complete ORFs. The relative positions of the amplified SCAR markers developed are indicated by a black line. GeneBank accession numbers: MH048872 (*Streptomyces* sp. VV/E1) and MH048873 (*Streptomyces* sp. VV/R4).

The *Streptomyces* sp. VV/E1 916 bp band (GenBank accession number MH048872) contained two partial open reading frames (ORF) (Fig 3A). The first one, extending from positions 1 to 401, exhibited a 61% amino acid identity with a TetR/AcrR family transcriptional regulator from *Streptomyces ipomoeae* 91–03 (accession number EKX60661.1), and lower amino acid identities with several other proteins from different actinobacterial strains also identified as transcriptional regulators belonging to that family. The second ORF, almost complete, stretched from positions 597 to 916 and might encode a protein that showed a 94% amino acid identity with a hypothetical protein from *S*. *ipomoeae* (accession number WP_048822038.1), and lower amino acid identities with many other hypothetical proteins from several *Streptomyces* species.

The *Streptomyces* sp. VV/R4 1862 bp band (GenBank accession number MH048873) contained 3 putative ORFs (Fig 3B). The first incomplete ORF, stretching from positions 1 to 509, encoded a protein with a very high amino acid identity (92%) to a hypothetical protein from *Streptomyces acidiscabies* (accession number WP_075662455.1). The second ORF was complete, and extended from positions 860 to 1306, encoding a protein with high similarity (87% amino acid identity) to a very short patch repair endonuclease from a *Streptomyce*s sp. strain (accession number WP_004939603.1). The third ORF was also truncated, stretching from positions 1331 to 1862. It encoded a protein that exhibited 69% amino acid identity to a DNA cytosine methyltransferase from *Streptomyces* sp. (accession number WP_053914715.1).

### Conversion of a RAPD marker into a SCAR marker

The nucleotide sequences of each of the cloned RAPD fragments were used to design pairs of SCAR primers targeting poorly conserved intergenic regions. After several attempts, a pair of primers, SCAR.E1_fw (positions 415 to 439; Fig 3A) and SCAR.E1_rv (positions 576 to 596; Fig 3A), were selected for *Streptomyces* sp. VV/E1 strain. Both primers were in the intergenic region located between the two ORFs detected. The SCAR.E1_fw and SCAR.E1_rv primers were designed with a 48.0% and 52.4% GC content, respectively, and they amplified a unique 182 bp PCR fragment from genomic DNA of the VV/E1 strain when annealing temperatures ranged from 60 to 65°C, indicating a high specificity of the amplification reaction.

Similarly, SCAR.R4_fw (positions 466 to 486; Fig 3B), and SCAR.R4_rv (positions 608 to 625; Fig 3B), were designed to amplify a specific 160 bp PCR fragment from VV/R4 strain genomic DNA when annealing temperatures ranged from 57 to 63°C. The SCAR.R4_fw primer had a 61.9% GC content and annealed near the 3’-end of ORF1. The SCAR.R4_rv primer had a 72.2% GC content and was located in the ORF1-ORF2 intergenic region (Fig 3B).

To test the specificity of the designed SCAR primer pairs, they were used to check if they amplified the genomic DNA of a total of 29 rhizosphere *Streptomyces* sp. strains (VV/R1 to VV/R29), and 21 endophytic actinobacterial strains, including 15 *Streptomyces* sp. isolates, 2 *Saccharopolyspora* sp. strains, and 3 *Micromonospora* sp. isolates [8] (S4 Fig) by conventional PCR. Only PCR products with the expected sizes were amplified from strains VV/E1 (182 bp) and VV/R4 (160 bp) (S4 Fig), with no other amplification in the other strains tested.

### Development of qPCR methods for the detection of *Streptomyces* sp. VV/E1 and *Streptomyces* sp. VV/R4 in vegetal samples

Once specific SCAR markers had been raised for both strains, rapid, sensitive, and specific methods for their detection were developed using quantitative real-time qPCR in plant material. The same primers used earlier were tested in a SYBR green qPCR system. The sensitivity of this system was evaluated for *Streptomyces* sp. VV/E1 and VV/R4 strains using purified DNA isolated from liquid culture mycelia.

**Fig 4:**
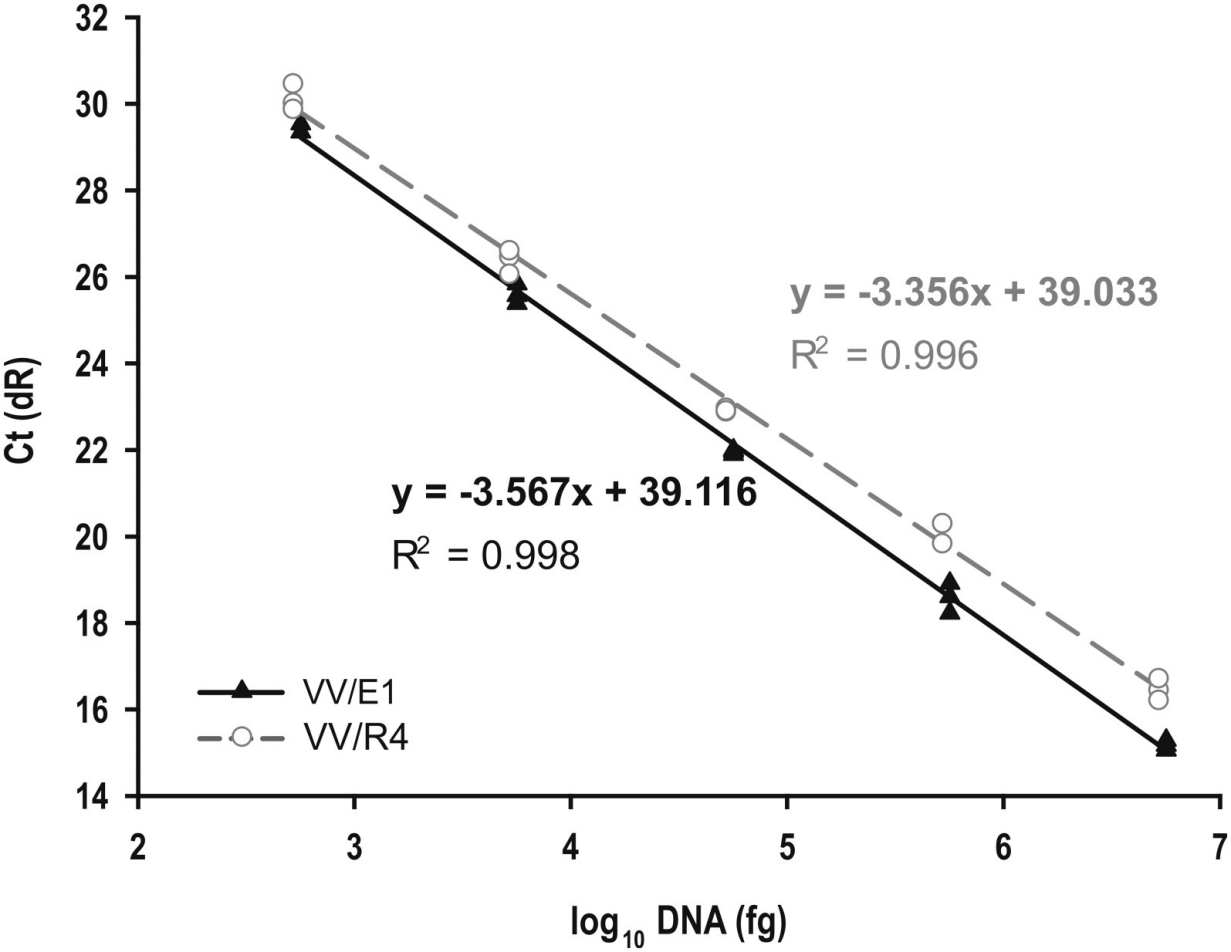
qPCR standard curves. Representative amplification curves obtained by plotting the mean *Ct* values with respect to the logarithm of genomic DNA quantity from *Streptomyces* sp. VV/E1 (black line) and *Streptomyces* sp. VV/R4 (grey line). Standard curves were generated with 10-fold serial dilutions of genomic DNA concentrations from 5.65 x 10^6^ fg to 56.5 fg (VV/E1 strain) or 5.2 x 10^6^ fg to 52.0 fg (VV/R4 strain) by SYBR green qPCR reactions.

Assays for *Streptomyces* sp. VV/E1 were performed using 10-fold serial dilutions of genomic DNA as templates. For the DNA amplification curve, the cycle threshold (*Ct*) values were converted into amount of DNA using the linear regression equation *y* = -3.567*x* + 39.116, where *x* is the logarithm of the DNA quantity (fg) and *y* is the *Ct* value. There was a high regression coefficient close to 1 (*R*^*2*^ = 0.998) and the amplification efficiency (E) was 90.7% (Fig 4). Similarly, assays for *Streptomyces* sp. VV/R4 strain were also carried out with 10-fold serial dilutions of genomic DNA as templates. The results allowed us to define the equation *y* = -3.356*x* + 39.033 (*R*^*2*^ = 0.996). The amplification efficiency (E) was 98.6% (Fig 4).

The LOQ was estimated to be 50.47 fg for VV/E1 and 72.65 fg for VV/R4 strains (S2 Fig).

To check that host (*Vitis vinifera*) DNA did not interfere with quantification of *Streptomyce*s sp. strain DNA over a wide range, additional standard curves for both strains were generated under the same conditions, but including grapevine genomic DNA extracted from wood (range from 1:1 to 1:100,000) of a non-inoculated grapevine plant. The linear regression equations obtained (S5 Fig) were very similar for each strain under both conditions. Furthermore, the corresponding slopes (S5 Fig) did not show significant differences, according to a Student’s t-test statistical analysis (*p* ≤ 0.05), which means that the presence of genomic DNA from grapevine wood did not have a significant impact on the results.

### Detection and quantification of *Streptomyces* sp. VV/E1 and VV/R4 strains in wood samples of inoculated grapevine plants

The plant inoculation efficacy of the different methods assayed was analysed 180 days after application, once the plants were uprooted. *Streptomyces* sp. VV/E1 and VV/R4 strains were quantified in plant material isolated from different zones (see Fig 1) according the inoculation method used in the batches analysed.

#### (i) Definition of the linear analysis models

Linear models were defined with the DNA quantification raw data (fg of DNA from both *Streptomyces* sp. strains detected in analysed samples), for every treatment (batch), and area analysed in order to explain the variability obtained (dependent variable). Three independent variables (treated as factors) were considered, as described in the materials and methods section. One variable is related to the inoculation methodology: we had control treatments (uninoculated plants, batches 1 and 6), inoculation by immersion (batches 2 and 3), and inoculation by injection (batches 4 and 5) treatments. The second factor was related to application of a booster treatment in batches 2, 3 and 6. The third factor was the plant area analysed. In the case of the control and immersion treatments, the RI and RS samples were analysed (Fig 1B). In grapevine plants inoculated by injection, four different zones were analysed (Z1/RI, Z2, Z3 and Z4; Fig 1A); Z1 was equivalent to the RI area in the control and immersion-treated grapevine plants.

#### (ii). Analysis of the inoculation efficiency by immersion

**Fig 5:**
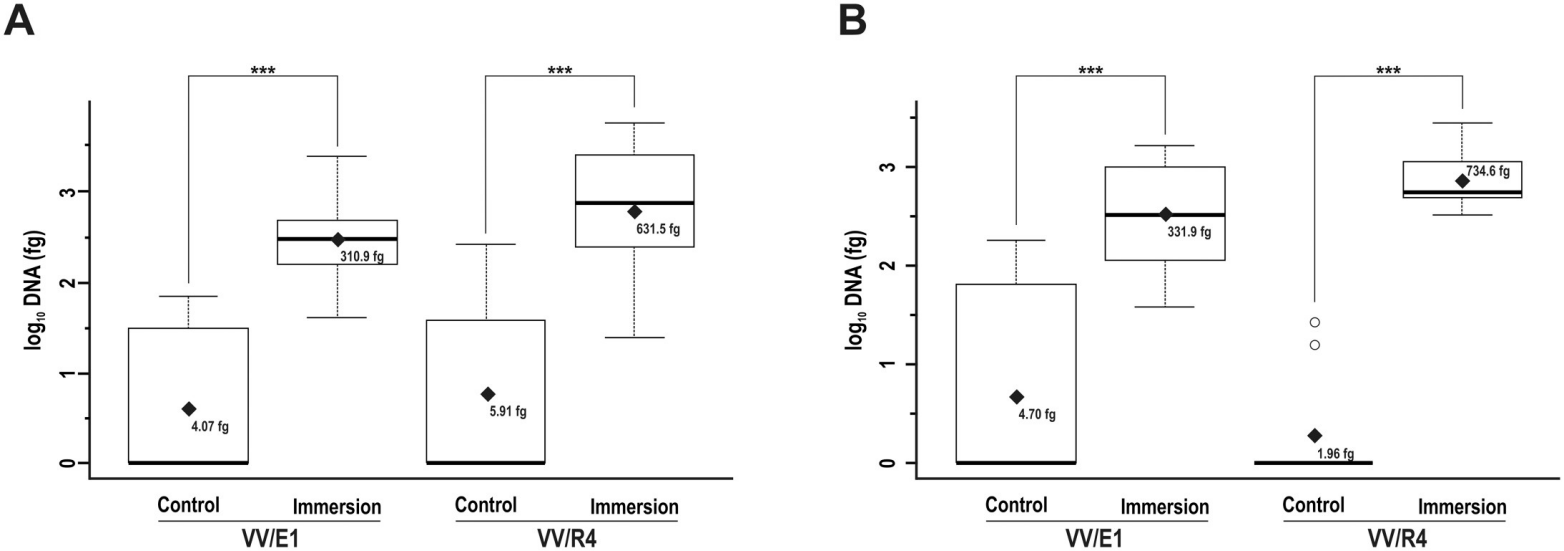
Quantification in RI and RS DNA samples from the *Streptomyces* sp. strains inoculated by immersion. Box plot showing the DNA levels from *Streptomyces* sp. VV/E1 and *Streptomyces* sp. VV/R4 strains in plants subjected to inoculation by immersion as compared to control plants in the (A) RI, and (B) RS areas. Black rhombuses show the average values (back transformed) for each treatment. Asterisks indicate existence of significant differences according the LSD-test performed: * *p* < 0.05; ** *p* < 0.01; *** *p* < 0.001. Circles correspond to data outliers (falling outside the Q1-Q3 range).

Once the linear model had been defined by considering the three independent variables treated as qualitative factors, the data analysis clearly indicated that the booster dose applied in batches 3, 5, and 6 was not efficient at promoting bacterial colonization of the root system (*p* values of 0.9190 and 0.5520 for VV/E1 and VV/R4, respectively). Accordingly, the second factor (application of a booster treatment on soil) was not taken into account in the final model. Therefore, batches with the same inoculation method, with and without booster treatment, were combined. Thus, further analysis was simplified by reducing treatments to just two: control samples (batches 1 and 6), and inoculation by immersion (batches 2 and 3), resulting in a larger sample size for each treatment and providing an increase in the robustness of the statistical analysis. Genomic DNA quantities for both VV/E1 and VV/R4 strains were measured at RI (Fig 5A) and RS (Fig 5B) in the samples. Data analysis showed the existence of significant differences (*p* < 0.001) in log_10_ DNA values for both RI and RS areas with respect to controls (batches 1 and 6). Mean values detected in RI were higher for the VV/R4 strain (631.45 fg DNA), compared to the VV/E1 strain (310.87 fg DNA). A similar trend was observed in the RS area: 734.62 fg of DNA detected for the VV/R4 strain and 331.87 fg of DNA for the VV/E1 strain. To check the correlation between the amount of DNA detected, and the corresponding number of cells, S1 Table should be consulted.

#### (iii). Analysis of the injection inoculation efficiency

**Fig 6:**
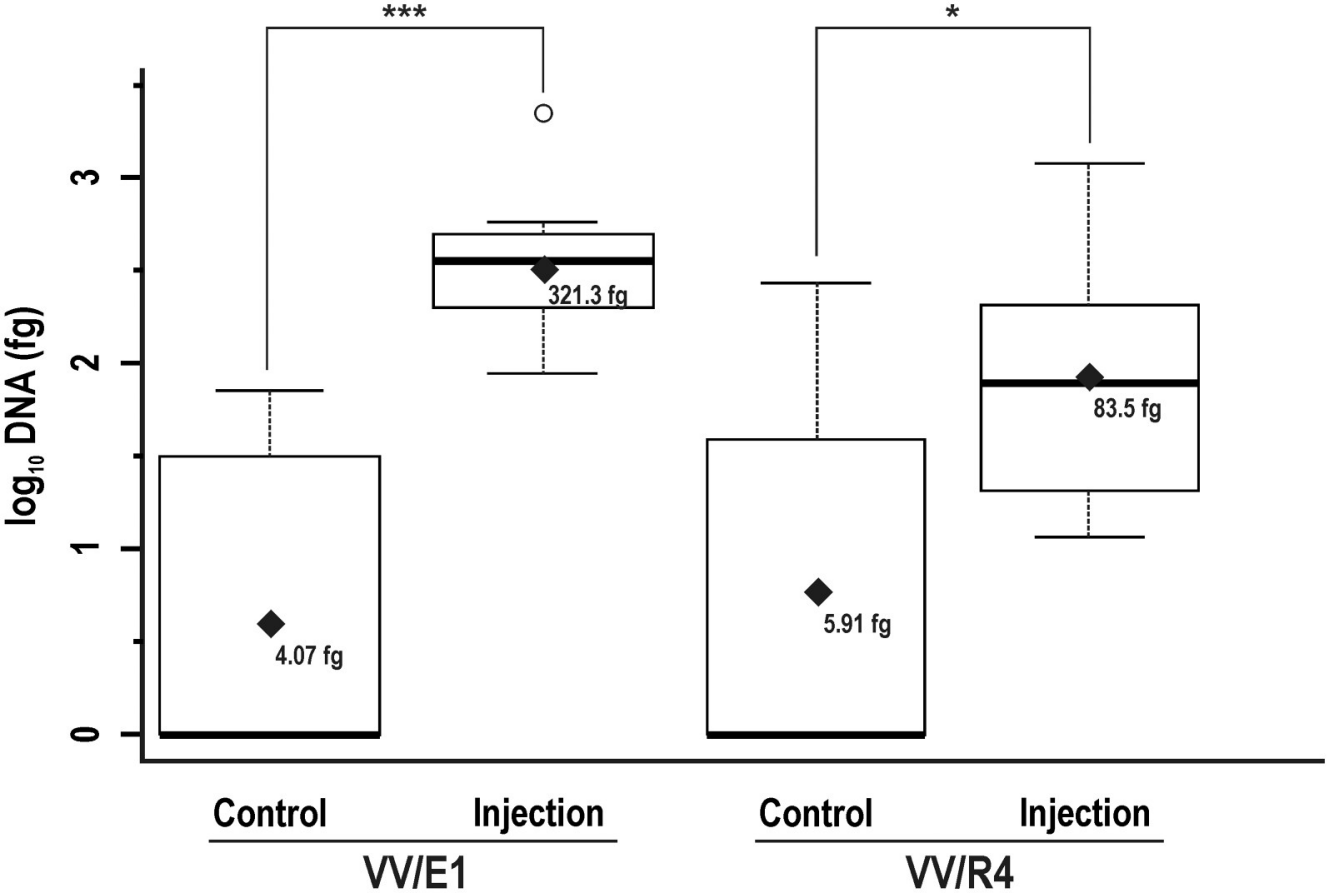
Quantification at RI of DNA from *Streptomyces* sp. strains inoculated by injection. Box plot showing the levels of DNA from *Streptomyces* sp. VV/E1 and *Streptomyces* sp. VV/R4 strains detected at RI in plants inoculated by injection, compared to the amount of DNA detected in control (untreated) plants. Black rhombuses correspond to average values (back transformed). Asterisks indicate significant differences according the LSD test: * *p* < 0.05; ** *p* < 0.01; *** *p* < 0.001. Circles correspond to outliers of the data (data falling outside the Q1-Q3 range).

Four different zones (Z1/RI to Z4) were analysed along the grapevine stem (Fig 1A). As in the previous treatment, a simplified model (according to *p* values of 0.6122 and 0.2106 for VV/E1 and VV/R4, respectively), in which the booster treatment was not taken into account, was used. Data analysis was carried out firstly by comparing injected and control plants at the RI point (or Z1/RI area in Fig 1A) (Fig 6). The log_10_ DNA amounts detected showed significant differences (*p* values of 7.096 x 10^−6^ and 2.996 x 10^−2^ for VV/E1 and VV/R4, respectively in the Z1/RI area) compared to the negative control (untreated plants). In this case, the mean DNA value, 321.3 fg, detected was clearly higher for the VV/E1 strain, as compared to 83.5 fg for the VV/R4 strain.

**Fig 7:**
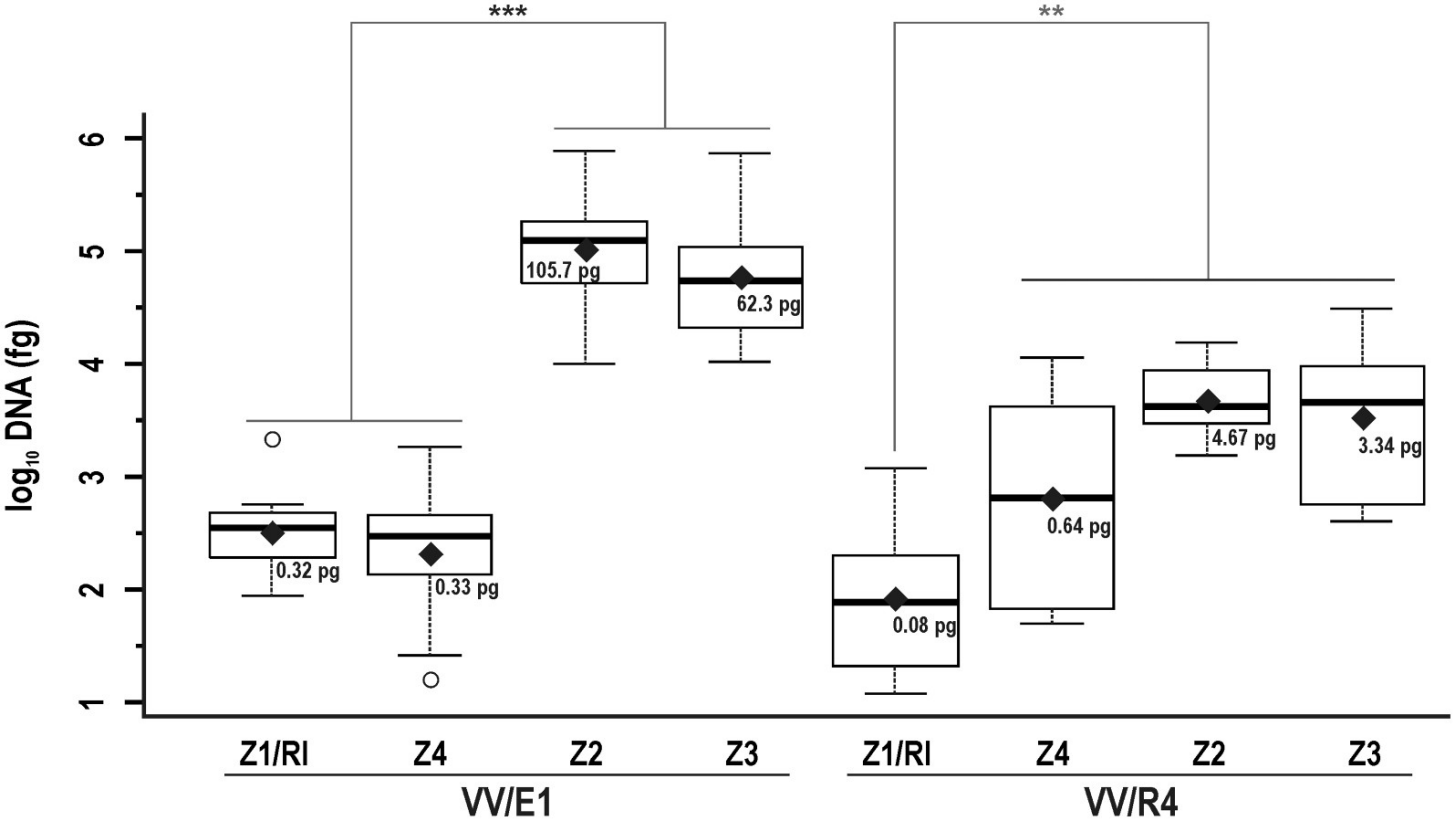
Quantification of *Streptomyces* sp. strain DNA at different zones of the rootstock inoculated by injection. Box plot corresponding to the DNA amounts of *Streptomyces* sp. VV/E1 and *Streptomyces* sp. VV/R4 strains detected in the different zones (Z1/R1 to Z4) analysed in the rootstock of plants inoculated by injection. Note that in this figure, DNA amounts are shown in pg. Black rhombuses correspond to average values (back transformed). Asterisks indicate significant differences according the LSD test: * *p* < 0.05; ** *p* < 0.01; *** *p* < 0.001. Circles correspond to data outliers (outside the Q1-Q3 range).

Secondly, we analysed the presence of DNA from *Streptomyces* sp. strains in the Z1/R1 to Z4 zones (Fig 1A) around the injection point (Fig 7). For the VV/E1 strain, significant differences were observed with respect to the distance from the inoculation point according to the LSD-test (*p* = 2.575 x 10^−12^). The amount of DNA detected in the distal areas Z1/RI (0.32 pg) and Z4 (0.33 pg) was low, whereas in the closer areas, Z2 (105.7 pg) and Z3, (62.3 pg) the amount of DNA was significantly higher when compared to Z1/RI and Z4 samples. As for the VV/R4 strain, there were significant differences (*p* = 1.821 x 10^−3^) between the Z1/RI (0.08 pg) and Z2-Z3-Z4 (values of 4.67, 3.34, and 0.64 pg, respectively) areas.

#### (iv) Comparison of the colonization rate of RI in plants inoculated by immersion or injection

**Fig 8:**
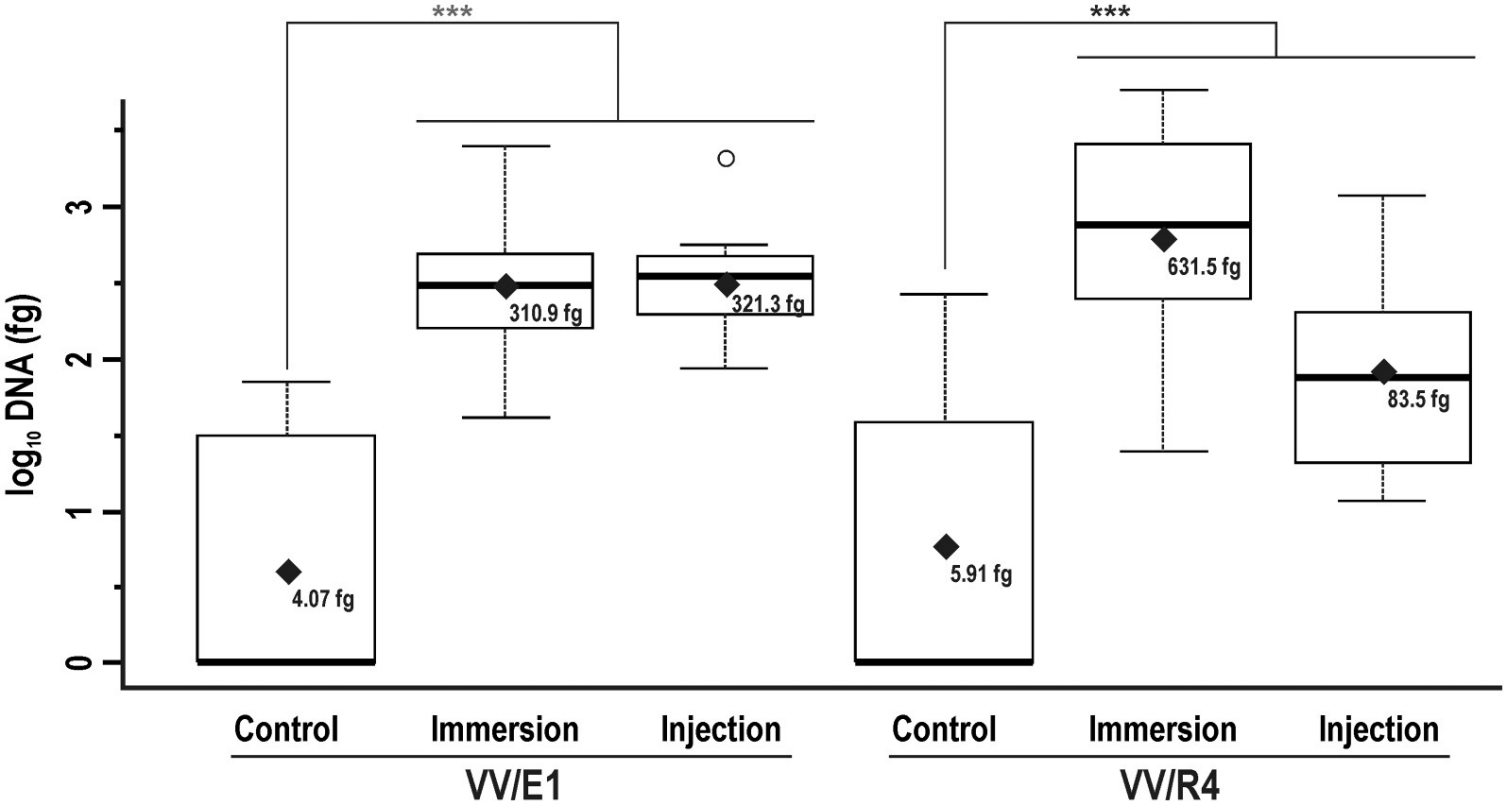
Comparison of the effectiveness of inoculation by immersion vs. injection at RI. Box plot showing DNA amounts at RI of *Streptomyces* sp. VV/E1 and *Streptomyces* sp. VV/R4 strains in plants inoculated by immersion or injection as compared to control (untreated) plants. Black rhombuses correspond to average values (back transformed) for each treatment. Asterisks indicate significant differences according to the LSD test: * *p* < 0.05; ** *p* < 0.01; *** *p* < 0.001. Circles correspond to data outliers (outside the Q1-Q3 range).

As we previously indicated, Z1/RI and RI were the only common areas to be analysed in plants inoculated by either immersion or injection (Fig 1). Consequently, it was of great interest to analyse the rate of bacterial colonization of this area in plants inoculated by both treatments. As in prior analysis, the full linear model was discarded, since no influence of the booster treatment in soil (*p* values of 0.601 and 0.205 for VV/E1 and VV/R4, respectively) was observed. The differences in log_10_ DNA amounts detected were significant (*p* = 7.598 x 10^−8^ for VV/E1, and *p* = 2.691 x10^-4^ for VV/R4) with respect to control plants (Fig 8). However, no significant differences between the two inoculation procedures could be detected. Indeed, VV/E1 strain mean values for both treatments were quite similar (310.87 and 331.33 fg DNA for immersion and injection, respectively). A remarkable difference was noticed for the VV/R4 strain, since higher DNA amounts were detected in plants inoculated using the immersion procedure (mean value of 631.45 fg of DNA) than in injected plants (83.48 fg of DNA) (Fig 8).

## Discussion

Bacteria belonging to the *Streptomyces* genus are well known as important secondary metabolite producers, excreting both antibacterial and antifungal compounds, and for their ability to control plant diseases [19–23]. The recent finding that the grapevine plant root system is home to numerous endophytic and rhizosphere *Streptomyces* species, and that some of these strains are effective at reducing the infection rate by fungi causing young grapevine decline (YGD) [8] in grafted grapevine plants, has suggested a putative use for these strains as biocontrol agents in vine nurseries [8].

However, this application was hampered by several factors including the absence of methods to specifically detect and quantify these strains in plant material, and effective, easy methods for introducing them into the grapevine plant root system to allow their colonization.

One of the objectives of the current study was to develop a simple molecular marker to detect *Streptomyces* sp. VV/E1 and VV/R4 strains in plant material. RAPDs are easy to develop as molecular markers, but lack of reproducibility blocks their reliable use. To improve their reproducibility, we converted RAPD amplicons into SCAR markers [24, 25] for VV/E1 and VV/R4 strains, raised against intergenic regions located inside the previously sequenced RAPDs. Since intergenic regions are poorly conserved between different species, or even strains, a SCAR marker developed from an intergenic region would likely be highly specific for a particular strain or species. In fact, the specificity of both SCAR markers was very high, since they did not allow amplification of, or cross-react with, genomic DNA from 50 other species of actinobacteria (mainly belonging to *Streptomyces* genus) that had been isolated as endophytic or rhizosphere strains from the root system of young grapevine plants [8]. However, we cannot totally rule out the existence of any taxon that could allow DNA amplification, for one, or both of the developed markers, although the robustness of the qPCR technology would allow us to discriminate that it was a false positive. On the one hand, there would be differences in the dissociation curve obtained in the qPCR assay, and on the other, there could even be differences in the size of the amplified fragment.

Although the use of SCAR markers allowed the detection of both strains from a low amount of only 30 ng genomic DNA, they did not allow an accurate quantification of the DNA detected. Accordingly, another goal of the current study was the development of a specific, sensitive qPCR assay for the detection and quantification of both strains in plant material. Given their high specificity, the same pair of primers used to amplify SCAR markers was used as specific primers for development of a qPCR protocol. The *Ct* values were linearly correlated with the concentration of target DNA for both strains, signifying that this methodology was suitable for qualitative and quantitative assays. The LOQ for both strains were quite low, indicating a good sensitivity of the methods. Unfortunately, there are hardly any studies on the quantification of *Streptomyces* strains by qPCR that allow comparison of the LOQ values obtained [26]. Since one of the objectives of this work was to quantify the presence of both strains in grapevine plants, we had to rule out the possible interference of grapevine genomic DNA in the qPCR assays. We tested broad concentration ratios (1:1 to 1:100,000) of DNA from both *Streptomyces* sp. strains against grapevine genomic DNA in qPCR experiments. The corresponding curve slopes did not show significant differences, which meant that the presence of grapevine genomic DNA did not interfere with the detection and quantification of the bacterial strains.

With the development of fast, reliable, accurate and specific qPCR methods for the detection of both strains in plant material, it was possible to analyse the colonization efficiency in the root system of young grapevine plants using two different inoculation methods: immersion and injection. Data analysis led us to several conclusions. First, that both strains could be detected inside the root system of non-inoculated (control) plants, although at a very low rate if we compared with the DNA amounts detected in inoculated plants. This result seems obvious for the VV/E1 strain since it was isolated as an endophytic strain. However, the VV/R4 strain was isolated as a rhizosphere strain. This data suggests that the line that separates the definition of a particular strain as endophytic or rhizosphere is very diffuse. Rhizosphere-established streptomycetes are well known to have different effects on plants [20–23]. Unfortunately, general knowledge about the impact of endophytic *Streptomyces* sp. strains on plant development is poorly understood, although it is clear that they can enhance the growth of some plants and help in the biocontrol of phytopathogens [22].

Since both strains were detected 180 days after their application and once the potted plants had completed a full growth cycle, it is clear that immersion of the root system in a bacterial suspension is a method that allows effective colonization. Both strains were detected at the RS as well as at the RI points. This indicates that the bacteria probably enter the root system through injuries produced when the roots were trimmed, before the bacterial application, although we cannot discard that the bacteria might enter through other microscopic injuries in the plants. RI detection also suggested an upward movement of the bacteria. Perhaps the bacteria move upward through the xylem vessels. Further experiments, including microscopy studies, could shed some light. The possibility that the bacteria might reach upper sections in the plant was not investigated. Current experiments should clarify this point. The behaviour of both strains was similar, although the VV/R4 strain was detected at higher levels than the VV/E1 strain at both RS and RI.

Injection of a bacterial suspension directly into the rootstock at a point 3 cm above the RI area was also effective at inducing colonization of the root system. The amount of genomic DNA detected at RI was clearly higher than that obtained for control (non-injected) plants. Nevertheless, the VV/E1 strain levels were higher than those detected for the VV/R4 strain (just the opposite result observed in the case of application by immersion). Taken together, immersion and injection data suggest that we cannot conclude that either of the strains is more adapted to an endophytic lifestyle. Analyses of the different Z1/RI, Z2, Z3, and Z4 samples around the injection point suggest that, from this point, the bacteria can move at least a short distance (2–3 cm) up and down the plants. Whether the bacteria could have travelled longer distances was not evaluated. The comparison of bacterial levels at the RI (Fig 8) suggest that for the VV/E1 strain, both methods exhibited a similar efficiency at colonizing the root system. However, immersion of the VV/R4 strain yielded a higher colonization rate than injection. We also analysed if the addition of a booster treatment, with direct addition of a bacterial suspension in the proximity of the root system, could reinforce the colonization rate of both strains previously inoculated by immersion or injection. According to our data, there was no improvement of colonization levels by either strain.

Since high densities of antagonistic *Streptomyces* are associated with plant disease suppression in some soils, some efforts have been made to apply the bacteria onto seeds (mainly by soaking the seeds in a suspension containing the biocontrol agent), or into soil at the highest possible density using different methods like mixing it with soil or into sowing furrows, or simply spreading it on the field using dripping systems [19, 22]. However, despite some success with this approach [27–30], the application of *Streptomyces* to soils as a biocontrol agent remains unreliable [31]. This failure to colonize the plant from the ground could be due to multiple factors among which is the competition with other microbial species existing in the root environment. Since the plants in this study were grown in a vegetal substrate, not in classical soil, it is possible that this is not the most appropriate substrate to allow for proper survival of the *Streptomyces* species. Therefore, we cannot rule out that the results obtained would have been different had we used a more traditional soil in the pots. However, our data indicate that *Streptomyces* sp. strains selected as biocontrol agents can be easily introduced into the root system of woody plants by simply soaking their trimmed root system in a bacterial suspension, or alternatively by direct injection into the vegetal tissues, thus providing a new methodology of inoculation. Taking into account these data, the potential for using endophytic *Streptomyces* to control plant disease is becoming a focus of research. Nonetheless, these studies are hampered by a poor understanding of the relationships between rhizosphere and endophytic populations, and how a *Streptomyces* strain becomes adapted to an endophytic way of life. It may be that the protected endophytic habitat minimizes the challenge of achieving successful colonization, which has proven to be difficult in competitive soil communities [31, 32].

Finally, we should emphasize that the current work shows that *Streptomyces* sp. strains isolated from grapevine root environment as putative biocontrol agents to fight fungal pathogens causing YGD, can be easily introduced into young grapevine plants by both immersion or injection, where they can establish and colonize the interior of the root system. Obviously, due to its ease of application, immersion should be the preferred option. This inoculation method could easily be applied in both nurseries, by the introduction of an additional inoculation step in the production of grafted plants, before young grapevine plants go on the market, as well as in cellars and vineyards, simply by placing the plants into a bacterial suspension prior to planting. Therefore, these biocontrol agents are a promising tool for diminishing the incidence of YGD in new plantations all over the world. These inoculation methods could also be potentially used for applying useful bacterial biocontrol strains to other vascular plants of great agronomical and economical interest like fruit trees (citrus, almonds or olive trees among others) affected by trunk diseases.

## Supporting information

S1 Fig RAPD amplification profiles. RAPD profiles generated by the amplification of rhizosphere (VV/R1 to VV/R5) and endophytic (VV/E1 to VV/E5) actinobacteria strains isolated from the root system of young grapevine plants using different primers: (A) OPA2, (B) OPA9, (C) OPA10, (D) P1, (E) P2, (F) P3, (G) P4, (H) P5, (I) R1, (J) R3, (K) SS1, (L) SS2 and (M) SS3. Specific bands selected for SCAR markers design are indicated by arrows. MW: Generuler 1 kb DNA Ladder Plus (Thermo Fisher Scientific). NC is the negative control.
(PDF)

S2 Fig Limit of quantification (LOQ) from *Streptomyces* sp. VV/E1 and VV/R4 strains. Coefficient of variation [*CV* = 100 x (*SD*/*mean value*)] of the back-calculated amounts of genomic DNA from (A) *Streptomyce*s sp. VV/E1 and (B) *Streptomyces* sp. VV/R4 by qPCR assays (5 replicates). Horizontal dashed lines correspond to *CV* = 35% and vertical dashed lines indicate the lowest quantity of DNA with a *CV* below 35% (obtained by interpolation). Grey symbols indicate the presence of negative (“non-detected”) replicates among samples.
(PDF)

S3 Fig Correlation between quantity of genomic DNA and number of cells. Standard curves obtained by plotting (A) the mean *Ct* values with respect to the logarithm of DNA copy number from purified and quantified PCR products. Standard curves obtained by plotting (B) the mean *Ct* values with respect to the logarithm of genomic DNA quantity (10-fold serial dilutions). (C) Correlation (logarithm scales) between genomic DNA quantity and the corresponding number of copies of each amplicon. *Streptomyces* sp. VV/E1 (black line) and *Streptomyces* sp. VV/R4 (grey line).
(PDF)

S4 Fig Specificity of SCAR primers. Absence of amplification from the genomic DNA of 50 actinobacterial endophytic and rhizosphere strains isolated from grapevine plants of the selected SCAR markers using SCAR primers (A) SCAR.E1_fw/SCAR.E1_rv and (B) SCAR.R4_fw/SCAR.R4_rv. Specific amplification bands were only detected for strains VV/E1 and VV/R4. MW: GeneRuler 1kb DNA Ladder Plus (Thermo Fisher Scientific). NC (negative control).
(PDF)

S5 Fig qPCR standard curves supplemented with grapevine genomic DNA. Standard curves obtained by SYBR Green qPCR using the primer sets that amplified the SCAR markers and generated with 10-fold serial dilutions of genomic DNA from (A) VV/E1 and (B) VV/R4 strains are shown in black lines. Grey lines correspond to the curves generated in the presence of grapevine genomic DNA. The final ratio of actinobacteria-to-grapevine DNA ranged from 1:1 to 1:100,000.
(PDF)

S1 Table Correlation between mean values of DNA amounts quantified by qPCR from *Streptomyces* sp. VV/E1 and VV/R4 strains and cell numbers. (DOCX)
