# Supplementary material for: Developing tools for evaluating inoculation methods of biocontrol Streptomyces sp. strains into grapevine plants
Source: PLoS One. 2019 Jan 24;14(1):e0211225. doi: 10.1371/journal.pone.0211225 (PMC6345443; doi:10.1371/journal.pone.0211225)
Supplement: S1 Fig — RAPD profiles generated by the amplification of rhizosphere (VV/R1 to VV/R5) and endophytic (VV/E1 to VV/E5) actinobacteria strains isolated from the root system of young grapevine plants using different primers: (A) OPA2, (B) OPA9, (C) OPA10, (D) P1, (E) P2, (F) P3, (G) P4, (H) P5, (I) R1, (J) R3, (K) SS1, (L) SS2 and (M) SS3. Specific bands selected for SCAR markers design are indicated by arrows. MW: Generuler 1 kb DNA Ladder Plus (Thermo Fisher Scientific). NC is the negative control. (PDF) [file pone.0211225.s001.pdf]

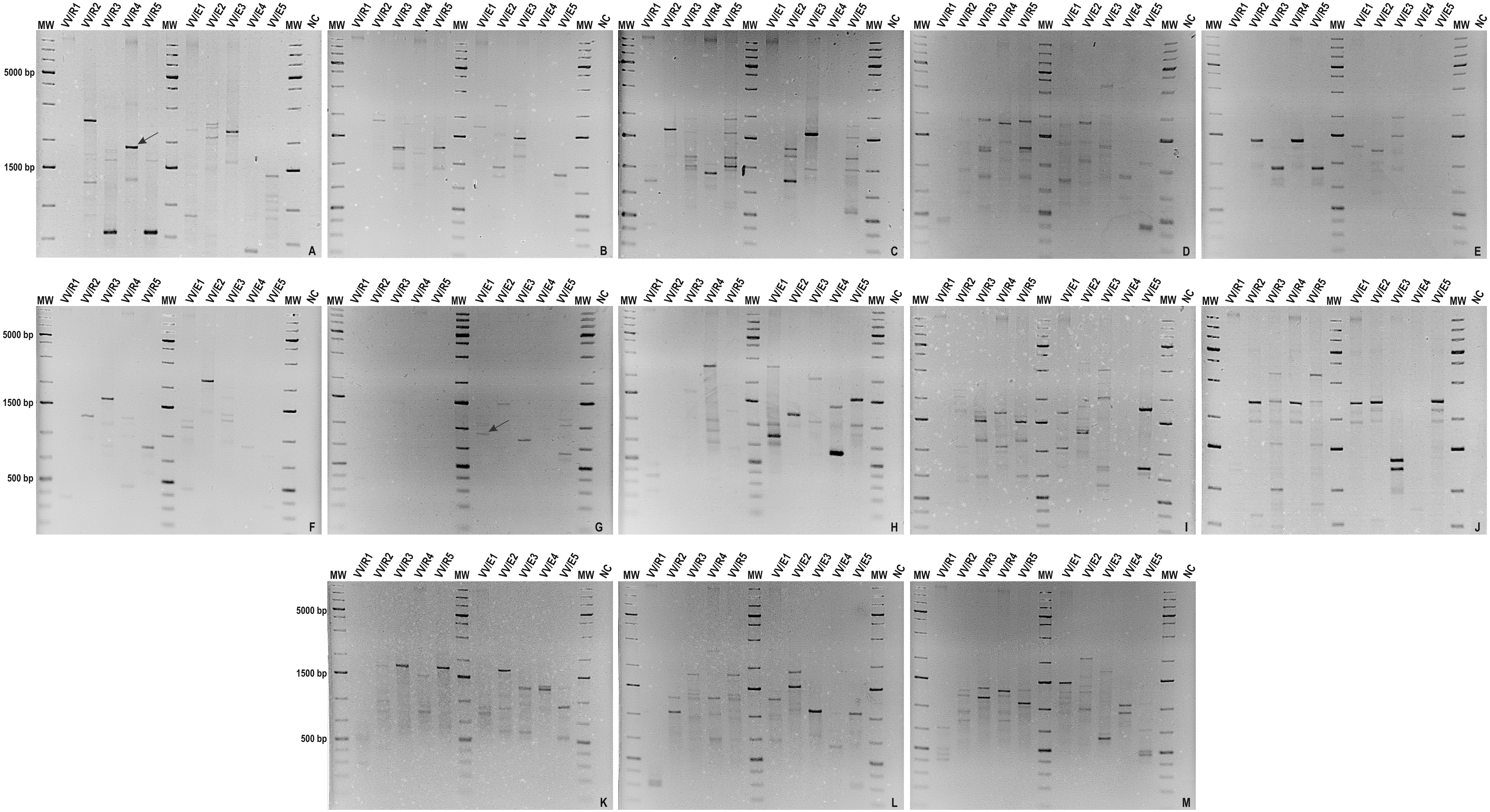

S1 Fig. RAPD amplification profiles. RAPD profiles generated by the amplification of rhizosphere (VV/R1 to VV/R5) and endophytic (VV/E1 to VV/E5) actinobacteria strains isolated from the root system of young grapevine plants using different primers: (A) OPA2, (B) OPA9, (C) OPA10, (D) P1, (E) P2, (F) P3, (G) P4, (H) P5, (I) R1, (J) R3, (K) SS1, (L) SS2 and (M) SS3. Specific bands selected for SCAR markers design are indicated by arrows. MW: Generuler 1 kb DNA Ladder Plus (Thermo Fisher Scientific). NC is the negative control.
