# Supplementary material for: Developing tools for evaluating inoculation methods of biocontrol Streptomyces sp. strains into grapevine plants
Source: PLoS One. 2019 Jan 24;14(1):e0211225. doi: 10.1371/journal.pone.0211225 (PMC6345443; doi:10.1371/journal.pone.0211225)
Supplement: S2 Fig — Coefficient of variation [CV = 100 x (SD/mean value)] of the back-calculated amounts of genomic DNA from (A) Streptomyces sp. VV/E1 and (B) Streptomyces sp. VV/R4 by qPCR assays (5 replicates). Horizontal dashed lines correspond to CV = 35% and vertical dashed lines indicate the lowest quantity of DNA with a CV below 35% (obtained by interpolation). Grey symbols indicate the presence of negative (“non-detected”) replicates among samples. (PDF) [file pone.0211225.s002.pdf]

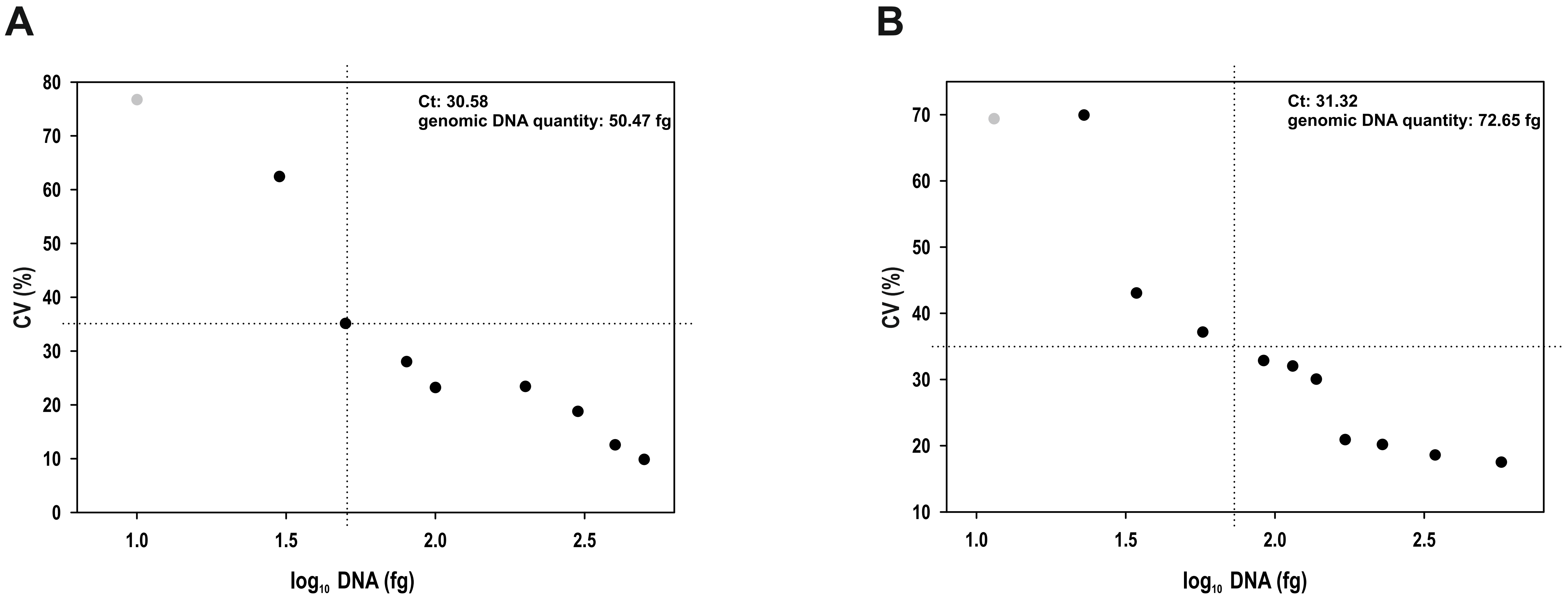

S2 Fig. Limit of quantification (LOQ) from *Streptomyces* sp. VV/E1 and VV/R4 strains. Coefficient of variation [*CV* = 100 x (*SD*/mean value)] of the back-calculated amounts of genomic DNA from (A) *Streptomyces* sp. VV/E1 and (B) *Streptomyces* sp. VV/R4 by qPCR assays (5 replicates). Horizontal dashed lines correspond to *CV* = 35% and vertical dashed lines indicate the lowest quantity of DNA with a *CV* below 35% (obtained by interpolation). Grey symbols indicate the presence of negative (“non-detected”) replicates among samples.
