# Supplementary material for: Developing tools for evaluating inoculation methods of biocontrol Streptomyces sp. strains into grapevine plants
Source: PLoS One. 2019 Jan 24;14(1):e0211225. doi: 10.1371/journal.pone.0211225 (PMC6345443; doi:10.1371/journal.pone.0211225)
Supplement: S3 Fig — Standard curves obtained by plotting (A) the mean Ct values with respect to the logarithm of DNA copy number from purified and quantified PCR products. Standard curves obtained by plotting (B) the mean Ct values with respect to the logarithm of genomic DNA quantity (10-fold serial dilutions). (C) Correlation (logarithm scales) between genomic DNA quantity and the corresponding number of copies of each amplicon. Streptomyces sp. VV/E1 (black line) and Streptomyces sp. VV/R4 (grey line). (PDF) [file pone.0211225.s003.pdf]

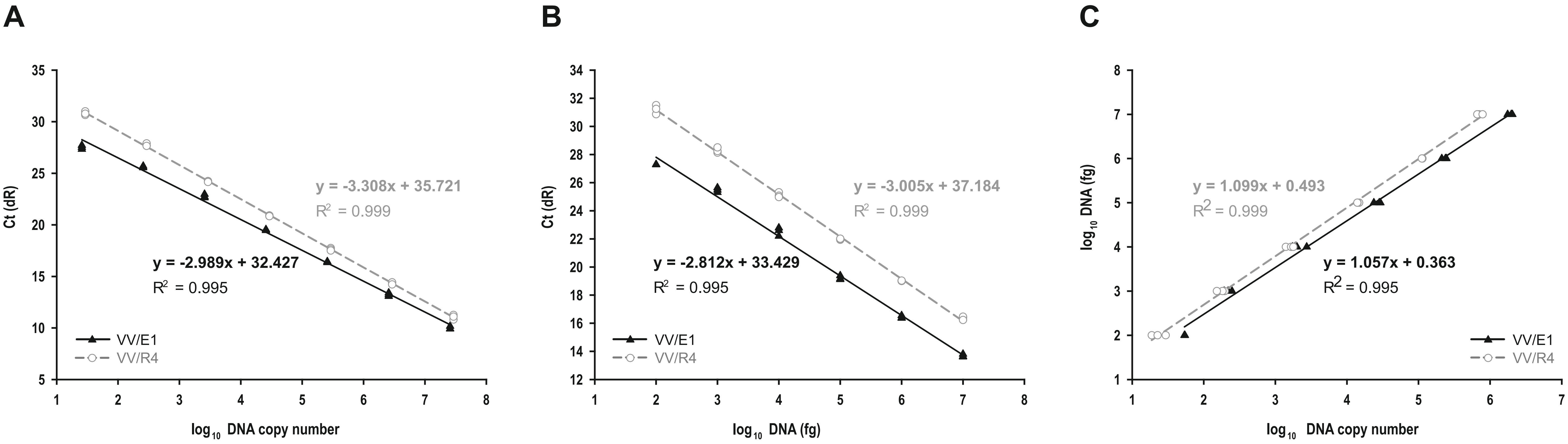

S3 Fig. Correlation between quantity of genomic DNA and number of cells. Standard curves obtained by plotting (A) the mean *Ct* values with respect to the logarithm of DNA copy number from purified and quantified PCR products. Standard curves obtained by plotting (B) the mean *Ct* values with respect to the logarithm of genomic DNA quantity (10-fold serial dilutions). (C) Correlation (logarithm scales) between genomic DNA quantity and the corresponding number of copies of each amplicon. *Streptomyces* sp. VV/E1 (black line) and *Streptomyces* sp. VV/R4 (grey line).
