# Supplementary material for: Developing tools for evaluating inoculation methods of biocontrol Streptomyces sp. strains into grapevine plants
Source: PLoS One. 2019 Jan 24;14(1):e0211225. doi: 10.1371/journal.pone.0211225 (PMC6345443; doi:10.1371/journal.pone.0211225)
Supplement: S4 Fig — Absence of amplification from the genomic DNA of 50 actinobacterial endophytic and rhizosphere strains isolated from grapevine plants of the selected SCAR markers using SCAR primers (A) SCAR.E1_fw/SCAR.E1_rv and (B) SCAR.R4_fw/SCAR.R4_rv. Specific amplification bands were only detected for strains VV/E1 and VV/R4. MW: GeneRuler 1kb DNA Ladder Plus (Thermo Fisher Scientific). NC (negative control). (PDF) [file pone.0211225.s004.pdf]

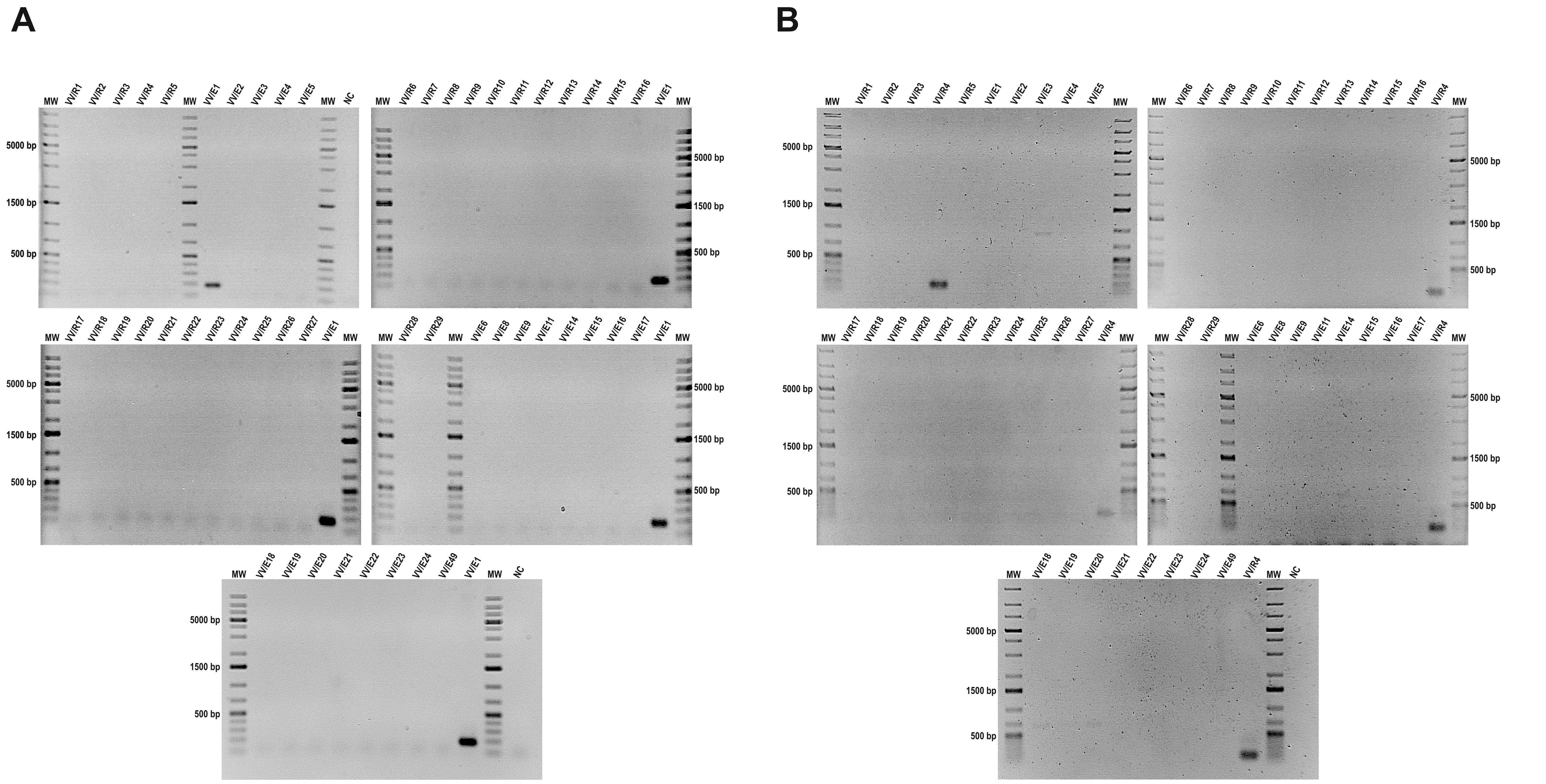

S4 Fig. Specificity of SCAR primers. Absence of amplification from the genomic DNA of 50 actinobacterial endophytic and rhizosphere strains isolated from grapevine plants of the selected SCAR markers by using SCAR primers (A) SCAR.E1_fw/SCAR.E1_rv and (B) SCAR.R4_fw/SCAR.R4_rv. Specific amplification bands were only detected for strains VV/E1 and VV/R4. MW: GeneRuler 1kb DNA Ladder Plus (Thermo Fisher Scientific). NC (negative control).
