# Supplementary material for: Developing tools for evaluating inoculation methods of biocontrol Streptomyces sp. strains into grapevine plants
Source: PLoS One. 2019 Jan 24;14(1):e0211225. doi: 10.1371/journal.pone.0211225 (PMC6345443; doi:10.1371/journal.pone.0211225)
Supplement: S5 Fig — Standard curves obtained by SYBR Green qPCR using the primer sets that amplified the SCAR markers and generated with 10-fold serial dilutions of genomic DNA from (A) VV/E1 and (B) VV/R4 strains are shown in black lines. Grey lines correspond to the curves generated in the presence of grapevine genomic DNA. The final ratio of actinobacteria-to-grapevine DNA ranged from 1:1 to 1:100,000. (PDF) [file pone.0211225.s005.pdf]

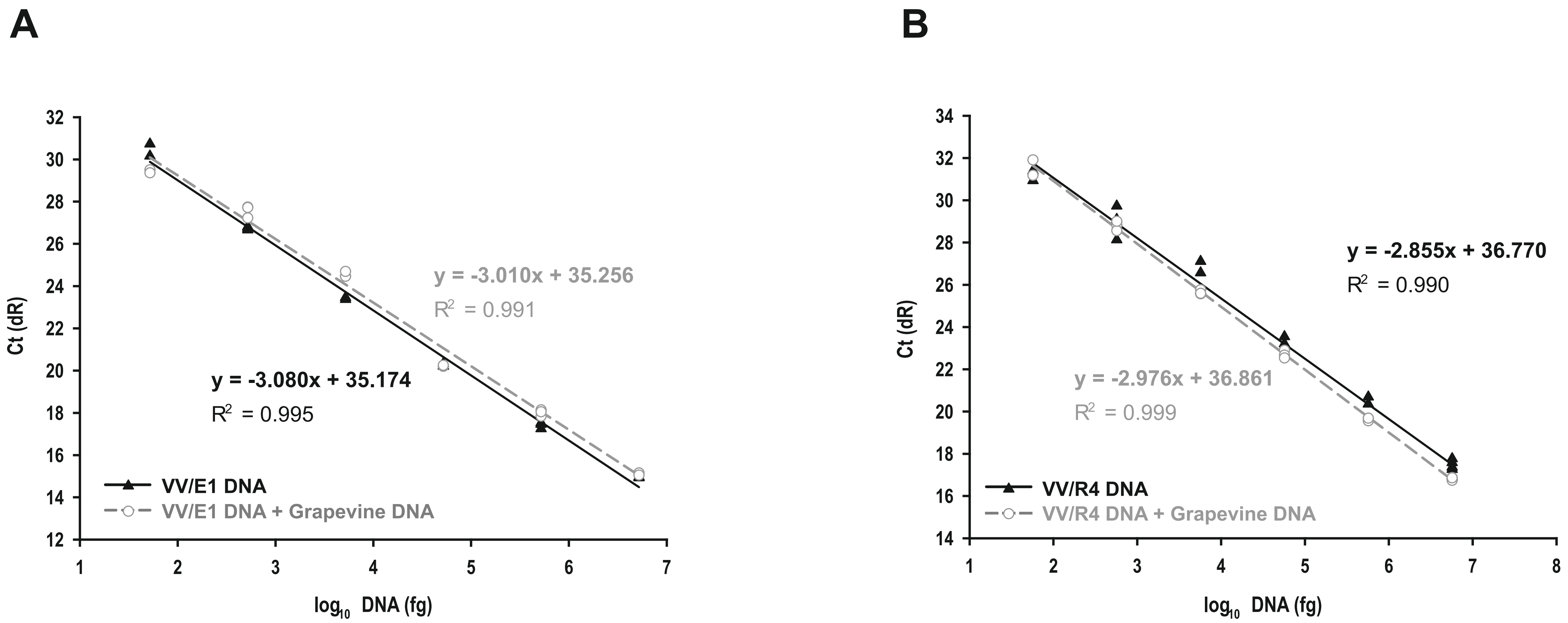

S5 Fig. qPCR standard curves supplemented with grapevine genomic DNA. Standard curves obtained by SYBR Green qPCR using the primer sets that amplified the SCAR markers and generated with 10-fold serial dilutions of genomic DNA from (A) VV/E1 and (B) VV/R4 strains are shown in black lines. Grey lines correspond to the curves generated in the presence of grapevine genomic DNA. The final ratio of actinobacteria-to-grapevine DNA ranged from 1:1 to 1:100,000.
