# Supplementary material for: Developing tools for evaluating inoculation methods of biocontrol Streptomyces sp. strains into grapevine plants
Source: PLoS One. 2019 Jan 24;14(1):e0211225. doi: 10.1371/journal.pone.0211225 (PMC6345443; doi:10.1371/journal.pone.0211225)
Supplement: S1 Table — (DOCX) [file pone.0211225.s006.docx]

| **S1 Table.** **Correlation between mean values of DNA amounts quantified by qPCR from *Streptomyces* sp. VV/E1 and VV/R4 strains and cell numbers.** | | | | |
| --- | --- | --- | --- | --- |
| **Strain** | **Treatment** | **Sample analysed *** | ***Streptomyces* sp. DNA (fg)** | ***Streptomyces* sp. cells/mg wood** |
| VV/E1 | *Control* | RI | 4.07 | 1.62 |
|  | *Immersion* |  | 310.9 | 26.5 |
|  | *Injection* |  | 321.3 | 31.2 |
| VV/R4 | *Control* |  | 5.91 | 1.76 |
|  | *Immersion* |  | 631.5 | 20.9 |
|  | *Injection* |  | 83.5 | 6.01 |
| VV/E1 | *Control* | RS | 4.70 | 2.35 |
|  | *Immersion* |  | 331.9 | 49.4 |
| VV/R4 | *Control* |  | 1.96 | 1.22 |
|  | *Immersion* |  | 734.6 | 67.5 |
| VV/E1 | *Injection* | Z1/RI | 321.3 | 31.2 |
|  |  | Z2 | 105719 | 6344 |
|  |  | Z3 | 60261 | 3735 |
|  |  | Z4 | 327.6 | 20.4 |
| VV/R4 | *Injection* | Z1/RI | 83.5 | 6.01 |
|  |  | Z2 | 4672 | 170.8 |
|  |  | Z3 | 3338 | 120.6 |
|  |  | Z4 | 642.5 | 20.9 |
| VV/E1 | *Control* | Z1/RI | 4.07 | 1.62 |
|  | *Immersion* |  | 310.9 | 26.5 |
|  | *Injection* |  | 321.3 | 31.2 |
| VV/R4 | *Control* |  | 5.91 | 1.76 |
|  | *Immersion* |  | 631.5 | 20.9 |
|  | *Injection* |  | 83.5 | 6.01 |
| * Root insertion point (RI), Root System (RS), Zone 1 (Z1), Zone 2 (Z2), Zone 3 (Z3), Zone 4 (Z4) analyzed in the rootstock as shown in Fig 1. Note that Z1 is equivalent to RI area for comparison purposes in plants subjected to inoculation by injection. | | | | |
